# Educational and economic disparities and risk factors associated with diabetes and impaired fasting glucose in Cambodia: analysis of a national population-based study

**DOI:** 10.7189/jogh.15.04251

**Published:** 2025-08-22

**Authors:** Rei Haruyama, Md Shafiur Rahman, Md Mahfuzur Rahman, Sam Ath Khim, Ada Moadsiri, Savina Chham, Srean Chhim, Hero Kol, Maly Phy

**Affiliations:** 1 Japan Institute for Health Security, Bureau of Global Health Cooperation, Tokyo, Japan; 2 Noncommunicable Disease Control Project, Japan International Cooperation Agency, Phnom Penh, Cambodia; 3 Graduate School of Health Innovation, Kanagawa University of Human Services, Kanagawa, Japan; 4 Research Center for Child Mental Development, Hamamatsu University School of Medicine, Shizuoka, Japan; 5 Division of Prevention, National Cancer Center Institute for Cancer Control, Tokyo, Japan; 6 Graduate School of Public Health, St. Luke's International University, Tokyo, Japan; 7 World Health Organization Cambodia, Phnom Penh, Cambodia; 8 Centre for Health Research and Policy Support, National Institute of Public Health, Phnom Penh, Cambodia; 9 Centre for Population, Family and Health, University of Antwerp, Antwerp, Belgium; 10 School of Public Health, National Institute of Public Health, Phnom Penh, Cambodia; 11 Julius Center for Health Sciences and Primary Care, University Medical Center Utrecht, Utrecht, the Netherlands; 12 Department of Public Health, Institute of Tropical Medicine Antwerp, Antwerp, Belgium; 13 Department of Preventive Medicine, Ministry of Health, Phnom Penh, Cambodia

## Abstract

**Background:**

In this study, we aimed to quantify the magnitude of educational and economic disparities and examine risk factors associated with diabetes and impaired fasting glucose in Cambodia.

**Methods:**

We used data from the 2023 STEPwise approach to noncommunicable risk factor surveillance to analyse 3660 participants aged 18–69 years. We quantified the extent of disparities using the regression-based slope index of inequality (SII) and relative index of inequality (RII). We used multi-level modified Poisson regression models to identify the potential risk factors.

**Results:**

Overall, the prevalence of diabetes and impaired fasting glucose was 6.4% (95% confidence interval (CI) = 5.6, 7.3) and 4.4% (95% CI = 3.6, 5.3). The magnitude of educational inequality in the prevalence of diabetes was significant, with the disease more concentrated among the non-educated population (SII = –7.6; 95% CI = –12.0, –3.3). Economic inequality in diabetes prevalence was less pronounced than education-based inequality at the national level (SII = –0.7; 95% CI = –4.5, 3.0); however, rural areas showed a concentration of diabetes among economically disadvantaged groups (SII = –4.7; 95% CI = –9.1, –0.3). Key factors associated with diabetes were advanced age ≥40 years, overweight (prevalence ratio (PR) = 1.4; 95% CI = 1.0, 2.0), obesity (PR = 1.7; 95% CI = 1.1, 2.5), comorbid hypertension (PR = 2.4; 95% CI = 1.8, 3.1), and daily alcohol consumption (PR = 2.0; 95% CI = 1.2, 3.3). Daily sugar-sweetened beverage consumption (PR = 1.8; 95% CI = 1.1, 3.1) also showed an increased risk of undiagnosed diabetes.

**Conclusions:**

The significant educational and economic disparities underscore the need for targeted interventions aimed at supporting non-educated and economically poor populations. Strengthening public health measures to address key risk factors, particularly alcohol and sugar-sweetened beverage consumption, is essential to curbing the growing burden of diabetes in Cambodia.

Diabetes poses a significant public health challenge globally, affecting 529 million people and responsible for 79.2 million disability-adjusted life years in 2021 [1]. The prevalence of diabetes has more than tripled from 151 million in 2000, and is projected to reach 1.31 billion by 2050 [1]. This rise is especially prominent in middle-income countries undergoing rapid demographic and epidemiological transitions [2]. Cambodia, a lower-middle-income country in Southeast Asia, is no exception to this trend. The prevalence of diabetes among people aged 25–64 years is reported to have increased from 2.9% in 2010 to 7.6% in 2023 [3].

Diabetes is a life-threatening disease that, if left untreated for a prolonged time, can lead to microvascular complications, including neuropathy, retinopathy, and nephropathy, as well as macrovascular complications such as cardiovascular diseases [4]. Prevention of modifiable risk factors and early detection, followed by appropriate management, can help delay these serious complications. In Cambodia, several studies have identified health system barriers in early detection and management of diabetes [5–9]. These include limited availability of diabetes services in public health facilities, shortage of medicines and test reagents, inadequate capacity of health providers, and high out-of-pocket payments required of the patients. There are other studies and reports reporting high prevalence of common behavioural risk factors related to diabetes, namely tobacco use, alcohol consumption, physical inactivity and unhealthy diet [3,10,11]. However, to date, the association between those risk factors and diabetes have not been well examined in Cambodia. One study carried out in two communities in 2005 reported female sex, older age, waist-to-hip ratio, and high blood pressure as significant predictors of individuals’ blood glucose concentration [12]. Another study conducted among people living with HIV in five provinces in 2017 reported low consumption of fruit as a single factor independently associated with diabetes [13]. However, the findings of these studies are not generalizable to the whole population. Other essential individual-level factors such as sugar-sweetened beverage (SSB) consumption and hypertension, as well as household-level factors (*e.g.* economic status) and community-level factors (*e.g.* urban residence) should also be considered [14–17].

Furthermore, while education and economic status are key factors that influence awareness, understanding and actions to prevent diabetes, the extent to which educational and economic disparities impact diabetes outcomes remains unclear. Therefore, we aimed to quantify the magnitude of educational and economic disparities and examine risk factors associated with diabetes and impaired fasting glucose (IFG) in the general population of Cambodia.

## METHODS

### Data source

We used data from the 2023 STEPwise approach to noncommunicable disease risk factor surveillance (STEPS survey) in Cambodia conducted by the Ministry of Health with support from the World Health Organization (WHO). The details of the survey have been described elsewhere [3]. In brief, this cross-sectional study was conducted in all 25 provinces across the country between June–July 2023. A multi-stage cluster sampling design was used to select a nationally representative sample of adults in Cambodia aged 18–69 years. At the first stage, 289 primary sampling units (PSUs) were randomly selected among 22 949 enumeration areas, each holding 100–200 households. Subsequently, 15 households per PSU were selected using simple random sampling from a list of households provided by the village chief. From each household, one adult member aged 18–69 years was randomly recruited to participate in the survey using a tablet-based application.

### Study participants

Among the households with adults aged 18–69 years, 4240 participated in the study. After excluding 307 adults with missing information on outcome and 273 with missing covariates, we included 3660 in this study (Figure S1 in the **Online Supplementary Document**).

### Outcomes

The outcomes of this study were the prevalence of diabetes and IFG. Since IFG represents a prediabetic state, examining IFG may inform timely interventions aimed at preventing the onset of diabetes, and thereby reducing the long-term health and economic burden [4].

In the Cambodia STEPS survey, participants were asked if they had ever been told by a doctor or other health worker that they had raised blood glucose or diabetes and if they had taken any medication or insulin for diabetes in the past two weeks, prescribed by a doctor or other health worker [3]. Additionally, participants had their fasting blood glucose (FBG) level measured using CardioCheck Plus analyser (PTS Diagnostics, Whitestown, Indiana, USA) after fasting for 12 hours [3]. Diabetes was defined as taking any medication or insulin in the past two weeks or FBG≥126 mg/dl (7 mmol/L) [3]. IFG was determined as FBG = 110 mg/dl (6.1 mmol/L)–125 mg/dl (6.9 mmol/L) [3]. In this study, we considered participants to have undiagnosed diabetes if they had diabetes but had never been told about it by a doctor or other health worker.

### Key exposures and covariates

For disparity analysis, we focused on two key exposures – the education level of participants and their household economic status. The Cambodia STEPS survey collected information on the highest level of education reported by the participants. These were categorised as no formal schooling, primary school (up to grade six), secondary school (up to grade nine), high school (up to grade 12) or higher education. Household economic status was categorised in quintiles based on per capita monthly income, calculated by dividing the total household income by the number of adult members in the household.

For the risk factor analysis, we analysed individual-level, household-level and community-level factors previously reported to be associated with diabetes [4,14–22]. Individual-level factors included age (18–29, 30–39, 40–49, 50–59, 60–69 years), sex (male, female), highest education level, marital status (currently married, others (never married, separated, divorced, widowed)), employment status (government employee, non-government employee, self-employed, unpaid (non-paid, students, homemaker, retired, unemployed)), body mass index (BMI) (underweight (<18.5 kg/m^2^), normal weight (18.5–<23.0 kg/m^2^), overweight (23.0–<27.5 kg/m^2^), obese (≥27.5 kg/m^2^)), smoking tobacco use (never, former user, current user), smokeless tobacco use (never, former user, current user), alcohol consumption (no, occasionally (<once per day), daily (≥once per day)), SSB consumption (no, occasionally (<once per day), daily (≥once per day)), fruits consumption (low (<1.5 servings on average per day), moderate (1.5–2.5 servings on average per day), adequate (≥2.5 servings on average per day)), vegetable consumption (low (<1.5 servings on average per day), moderate (1.5 to <2.5 servings on average per day), adequate (≥2.5 servings on average per day)), physical activity (low (<150 minutes of moderate-intensity activity per week or equivalent), adequate (≥150 minutes of moderate-intensity activity per week or equivalent)), and comorbid hypertension (no, yes). We used the BMI cutoff points for Asian populations (≥23 kg/m^2^), as previous research in Cambodia has linked this threshold to an increased risk of hypertension, diabetes, and hypercholesterolemia, highlighting its relevance as a potential trigger for public health interventions [23,24]. SSBs included carbonated soft drinks and sugar-sweetened drinks, including sports drinks, energy drinks, fruit juices, and other sugar-sweetened milk, teas and coffees [3]. We considered participants hypertensive if the average of the second and third measurement of their systolic blood pressure was ≥140 mm Hg or diastolic blood pressure was ≥90 mm Hg, or if they reported currently taking antihypertensive medication [25].

We used household economic status as household-level factor. Community-level factors included participant’s place of residence (rural or urban) and region (Central Plain (Kampong Cham, Tbong Khmum, Kandal, Phnom Penh, Prey Veng, Svay Rieng, and Takeo), Tonle Sap (Banteay Meanchey, Battambang, Kampong Chhnang, Kampong Thom, Pursat, Siem Reap, Oddar Meanchey, and Pailin), Coastal and Sea (Kampot, Koh Kong, Preah Sihanouk, and Kep), Plateau and Mountains (Kampong Speu, Kratie, Mondulkiri, Preah Vihear, Ratanakiri, and Stung Treng)) based on the 2019 census [26].

### Statistical analysis

We described the background characteristics of the study participants using percentage distributions. We calculated the prevalence of diabetes and IFG as rates per 100 people with 95% confidence intervals (CI). We used the χ^2^ test to evaluate whether the prevalence of diabetes and IFG significantly differed across the categories of independent variables. All statistical analyses accounted for sampling weights to ensure population representativeness.

We examined the magnitude of educational and economic disparities in the prevalence of diabetes and IFG using both absolute and relative measures of inequality. For the absolute measure, we used a regression-based slope index of inequality (SII), which quantifies the difference in estimated indicator values between the most-advantaged and most-disadvantaged subgroups [27]. An SII value of zero indicates that there is no inequality, while a negative value indicates that the outcome is more prevalent among the disadvantaged subgroup and vice-versa. Similarly, for the relative measure, we used a regression-based relative index of inequality (RII). The RII represents the ratio of estimated indicator values of the most advantaged to the most disadvantaged subgroup [27]. An RII value of one indicates that there is no inequality, while an RII<1 indicates that the outcome is more prevalent among the most disadvantaged group and vice-versa.

We used multi-level modified Poisson regression models with a random intercept term at the PSU-level to identify potential risk factors for diabetes and IFG. We chose a modified Poisson regression model over a logistic regression model because the outcomes were rare, and it provides unbiased estimates [28,29]. Model 1 was an unadjusted model. We adjusted Model 2 for individual-level factors and Model 3 for household and contextual factors. We reported prevalence ratios (PR) together with 95% CIs. In addition, we conducted a sensitivity analysis excluding participants who had been previously diagnosed with diabetes and therefore were aware of their condition before this survey to examine the factors associated with undiagnosed diabetes (n = 369).

We performed data management and statistical analyses using Stata SE, version 18 (StataCorp, College Station, Texas, USA). We considered *P*-value <0.05 statistically significant.

## RESULTS

### Background characteristics of study participants

Among the 3660 participants, 68.0% were aged ≥40 years and 64.8% were women (**Table 1**). Nearly 60.0% of the participants had no formal schooling. 69.1% were married, and 81.1% were either self-employed or unpaid. While the majority never used tobacco products and had high levels of physical activity, over half of the participants were overweight, and one-fourth had hypertension (26.4%). 62.1% of the participants resided in rural areas.

**Table 1:** Prevalence of diabetes and impaired fasting glucose among adults in Cambodia

Variable name	N (%)	IFG prevalence (95% CI)	*P*-value	Diabetes prevalence (95% CI)	*P*-value
Overall	3660 (100.0)	4.4 (3.6, 5.3)		6.4 (5.6, 7.3)	
Age in years			<0.001		<0.001
*18–29*	465 (13.6)	1.4 (0.6, 3.3)		0.9 (0.4, 2.1)	
*30–39*	704 (20.5)	4.5 (2.8, 7.2)		3.3 (2.1, 5.3)	
*40–49*	737 (21.5)	5.8 (4.0, 8.2)		7.9 (5.9, 10.6)	
*50–59*	819 (23.9)	8.9 (6.6, 11.9)		16.7 (13.6, 20.2)	
*60–69*	703 (20.5)	8.5 (6.2, 11.5)		20.9 (17.3, 25.0)	
Sex			0.111		0.399
*Male*	1289 (35.2)	4.4 (3.3, 5.9)		5.4 (4.3, 6.8)	
*Female*	2371 (64.8)	4.3 (3.4, 5.5)		7.5 (6.4, 8.8)	
Highest educational level			0.001		0.050
*No formal schooling*	2142 (58.5)	5.5 (4.3, 7.0)		8.9 (7.5, 10.5)	
*Primary school completed*	678 (18.5)	4.1 (2.4, 6.8)		3.7 (2.6, 5.1)	
*Secondary school completed*	497 (13.6)	3.2 (2.0, 5.0)		6.0 (4.2, 8.5)	
*High school or higher education completed*	343 (9.4)	2.8 (1.4, 5.5)		3.5 (2.2, 5.6)	
Marital status			0.909		0.063
*Currently married*	2528 (69.1)	4.5 (3.7, 5.6)		7.1 (6.1, 8.3)	
*Others (never married/separated divorced/widowed)*	1132 (30.9)	4.0 (2.6, 6.0)		4.7 (3.6, 6.2)	
Employment status			<0.001		0.103
*Government employee*	128 (3.5)	10.8 (5.5, 20.0)		5.1 (2.6, 9.7)	
*Non-government employee*	566 (15.5)	4.1 (2.7, 6.2)		4.7 (3.2, 7.0)	
*Self-employed*	2162 (59.1)	4.5 (3.4, 5.8)		6.4 (5.3, 7.6)	
*Unpaid (non-paid, students, homemakers, retirees, unemployed)*	804 (22.0)	3.0 (2.1, 4.3)		9.0 (7.0, 11.6)	
BMI			<0.001		<0.001
*Underweight (<18.5 kg/m^2^)*	339 (9.3)	2.3 (1.0, 5.0)		2.1 (1.0, 4.3)	
*Normal (18.5–<23 kg/m^2^)*	1436 (39.2)	3.2 (2.3, 4.5)		4.8 (3.8, 6.0)	
*Overweight (23.0–<27.5 kg/m^2^)*	1295 (35.4)	4.8 (3.3, 6.8)		8.2 (6.7, 10.0)	
*Obese (≥27.5 kg/m^2^)*	590 (16.1)	9.5 (6.7, 13.3)		12.0 (9.0, 15.9)	
Smoking tobacco product use			0.326		0.666
*Never*	2752 (75.2)	4.3 (3.4, 5.4)		6.3 (5.4, 7.4)	
*Former user*	347 (9.5)	4.7 (2.8, 7.6)		7.6 (5.1, 11.1)	
*Current user*	561 (15.3)	4.5 (2.7, 7.6)		6.2 (4.3, 8.7)	
Smokeless tobacco product use			0.100		0.213
*Never*	3291 (89.9)	4.3 (3.5, 5.3)		6.1 (5.2, 7.0)	
*Former user*	98 (2.7)	8.3 (3.2, 20.1)		9.4 (5.1, 16.5)	
*Current user*	271 (7.4)	4.8 (2.6, 8.8)		14.7 (9.9, 21.2)	
Alcohol consumption			<0.001		<0.01
*No*	963 (26.3)	5.7 (4.0, 8.2)		8.1 (6.4, 10.2)	
*Occasionally (<once per day)*	2567 (70.1)	4.0 (3.1, 5.1)		5.7 (4.8, 6.8)	
*Daily (≥once per day)*	130 (3.6)	6.2 (3.2, 11.7)		13.8 (8.6, 21.5)	
Sugar-sweetened beverage consumption			<0.001		<0.05
*No*	793 (21.7)	5.6 (3.9, 8.0)		11.9 (9.4, 15.0)	
*Occasionally (<once per day)*	1845 (50.4)	4.8 (3.7, 6.3)		5.2 (4.2, 6.4)	
*Daily (≥once per day)*	1022 (27.9)	3.0 (2.0, 4.4)		5.7 (4.4, 7.4)	
Fruits consumption			0.649		0.095
*Low (<1.5 servings per day)*	2672 (73.4)	4.0 (3.1, 5.1)		6.2 (5.3, 7.2)	
*Moderate (1.5–2.5 servings per day)*	434 (11.9)	4.5 (2.7, 7.4)		7.4 (5.1, 10.8)	
*Adequate (≥2.5 servings per day)*	534 (14.7)	5.6 (3.7, 8.3)		6.3 (4.4, 8.9)	
Vegetable consumption			0.368		0.078
*Low (<1.5 servings per day)*	1676 (45.8)	3.9 (2.8, 5.6)		6.2 (5.1, 7.5)	
*Moderate (1.5–2.5 servings per day)*	876 (23.9)	4.4 (3.0, 6.3)		5.3 (4.0, 7.1)	
*Adequate (≥2.5 servings per day)*	1108 (30.3)	4.9 (3.6, 6.6)		7.6 (6.0, 9.5)	
Physical activity			<0.05		0.586
*Low (<150 min of moderate-intensity activity per week or equivalent)*	226 (6.2)	1.9 (0.9, 3.9)		7.9 (4.9, 12.6)	
*Adequate (≥150 min of moderate-intensity activity per week or equivalent)*	3434 (93.8)	4.5 (3.7, 5.5)		6.3 (5.5, 7.3)	
Comorbid hypertension			<0.001		<0.001
*No*	2694 (73.6)	3.7 (2.9, 4.8)		3.9 (3.2, 4.6)	
*Yes*	966 (26.4)	7.8 (5.8, 10.3)		19.8 (16.4, 23.7)	
Household income in quantiles			0.336		<0.05
*Poorest*	732 (20.0)	3.5 (2.3, 5.3)		9.9 (7.6, 12.9)	
*Poorer*	743 (20.3)	6.0 (3.9, 9.1)		5.2 (3.8, 7.0)	
*Middle*	791 (21.6)	3.7 (2.5, 5.5)		4.7 (3.5, 6.3)	
*Richer*	686 (18.7)	4.3 (2.9, 6.4)		5.5 (4.1, 7.4)	
*Richest*	708 (19.3)	4.4 (2.6, 7.3)		6.9 (5.0, 9.4)	
Place of residence			<0.001		<0.05
*Rural*	2274 (62.1)	4.1 (3.2, 5.3)		5.6 (4.7, 6.7)	
*Urban*	1386 (37.9)	4.9 (3.6, 6.7)		8.3 (6.8, 10.1)	
Region			<0.05		0.372
*Central Plain*	1758 (48.0)	4.4 (3.3, 5.8)		7.2 (6.0, 8.7)	
*Tonle Sap*	1196 (32.7)	4.7 (3.3, 6.7)		6.6 (5.2, 8.3)	
*Coastal and sea*	216 (5.9)	3.9 (2.1, 7.3)		3.8 (2.1, 6.8)	
*Plateau and mountains*	490 (13.4)	3.7 (2.2, 6.4)		5.1 (3.5, 7.3)	

BMI – body mass index, CI – confidence interval, IFG – impaired fasting glucose

### Prevalence of diabetes and IFG

Overall, the prevalence of diabetes was 6.4% (95% CI = 5.6, 7.3) and IFG 4.4% (95% CI = 3.6, 5.3) (**Table 1**). The prevalence of diabetes increased with advancing age and higher BMI. A higher prevalence was also observed among participants reporting daily alcohol consumption (compared to occasional consumption), with comorbid hypertension (compared to those without), from the poorest households (compared to other income quintiles), and residing in urban areas (compared to rural areas). Participants reporting no SSB consumption also exhibited a higher prevalence than those with occasional or daily consumption.

**Figure 1:**
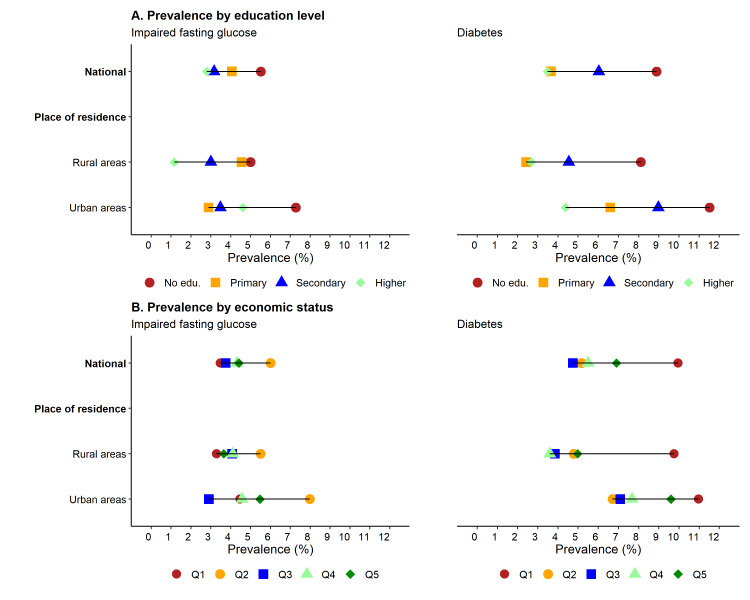
Prevalence of diabetes and impaired fasting glucose by education level of participants and their household economic status. **Panel A.** Prevalence by education level. **Panel B.** Prevalence by economic status.

The prevalence of both diabetes and IFG appeared to be the highest among participants with no formal education in both rural and urban areas (**Figure 1**, Panel A; Table S1 in the **Online Supplementary Document**). Similarly, the prevalence of diabetes appeared to be highest among participants from the poorest households across rural (9.8%; 95% CI = 5.9, 15.8) and urban settings (11.0%; 95% CI = 3.6, 28.9) (**Figure 1**, Panel B; Table S2 in the **Online Supplementary Document**).

### Magnitude of educational disparities in the prevalence of diabetes and IFG

**Figure 2:**
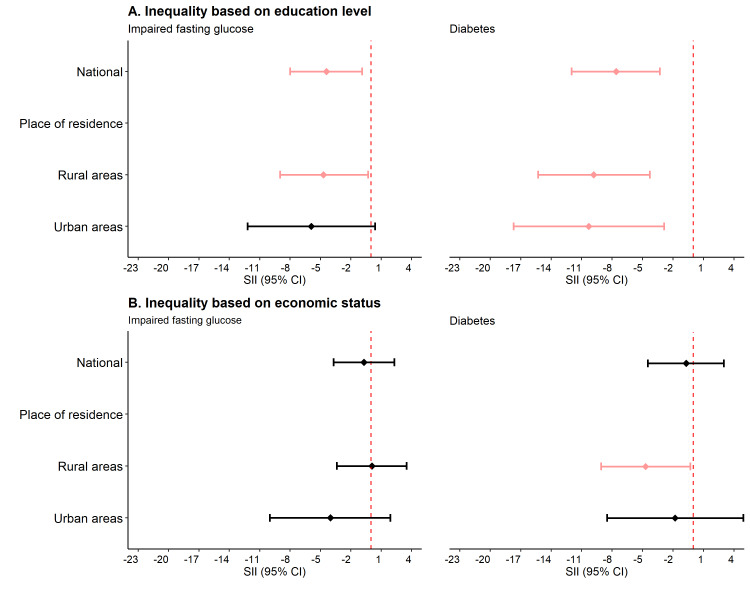
Absolute inequality in the prevalence of diabetes and impaired fasting glucose. **Panel A.** Inequality based on education level. **Panel B.** Inequality based on economic status. CI – confidence intervals, SII – slope index of inequality.

**Figure 3:**
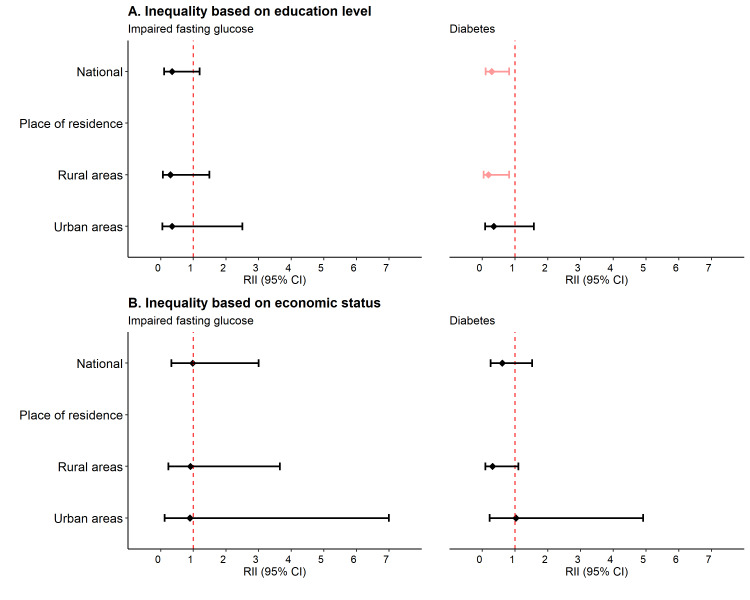
Relative inequality in the prevalence of diabetes and impaired fasting glucose. **Panel A.** Inequality based on education level. **Panel B.** Inequality based on economic status. CI – confidence intervals, RII – relative index of inequality.

At the national level, the prevalence of diabetes and IFG was 7.6 percentage points (SII = –7.6; 95% CI = –12.0, –3.3) and 4.4 percentage points (SII = –4.4; 95% CI = –8.0, –0.9) higher, respectively, among the non-educated population compared to the higher-educated population (**Figure 2**, Panel A; Table S3 in the **Online Supplementary Document**). The magnitude of such absolute inequality was even higher when examining rural and urban areas separately. The relative inequality analysis showed that, at the national level, a more educated population were 71% less likely (RII = 0.3; 95% CI = 0.1, 0.8) to have diabetes compared to the non-educated population (**Figure 3**, Panel A; Table S3 in the **Online Supplementary Document**).

### Magnitude of economic disparities in the prevalence of diabetes and IFG

At the national level, the absolute economic inequalities in the prevalence of diabetes and IFG were non-significant (for diabetes SII = –0.7; 95% CI = –4.5, 3.0 and IFG SII = –0.7; 95% CI = –3.7, 2.3) (**Figure 2**, Panel B; Table S3 in the **Online Supplementary Document**). However, in rural areas, a significant absolute inequality was observed; the prevalence of diabetes was 4.7 percentage points higher among the poorest households compared to the richest households (SII = –4.7; 95% CI = –9.1, –0.3). Similar findings were observed for relative inequality; participants from the richest households were 69% less likely to have diabetes than their poorest counterparts, although the result was not statistically significant (RII = 0.3; 95% CI = 0.1, 1.1) in rural areas (**Figure 3**, Panel B; Table S3 in the **Online Supplementary Document**).

### Factors associated with diabetes and IFG

After adjusting for individual, household, and contextual-level factors, age ≥40 years and obesity were associated with diabetes and IFG (**Table 2**). In addition to these two factors, secondary school completion (PR = 1.6; 95% CI = 1.1, 2.3), overweight (PR = 1.4; 95% CI = 1.0, 2.0), daily alcohol consumption (PR = 2.0; 95% CI = 1.2, 3.3), comorbid hypertension (PR = 2.4; 95% CI = 1.8, 3.1), and poor economic status (PR = 1.6; 95% CI = 1.1, 2.2) showed an increased risk of diabetes. In the sensitivity analysis, which excluded participants with a prior diabetes diagnosis, the associations with secondary school completion were no longer statistically significant. On the other hand, non-government employee and unpaid status (PR = 3.0; 95% CI = 1.1, 8.3 and PR = 3.1; 95% CI = 1.0, 9.0, respectively) and daily SSB consumption (PR = 1.8; 95% CI = 1.1, 3.1) showed significant associations with undiagnosed diabetes (**Table 3**).

**Table 2:** Risk factors for diabetes and impaired fasting glucose

Variables	Impaired fasting glucose, prevalence ratio (95% CI)			Diabetes, prevalence ratio (95% CI)		
	Model 1*	Model 2*	Model 3*	**Model 1**	**Model 2**	**Model 3**
Age in years						
*18–29*	ref	ref	ref	ref	ref	ref
*30–39*	3.3 (1.5, 7.5)†	2.9 (1.3, 6.4)†	2.9 (1.3, 6.6)†	3.6 (1.4, 9.2)†	4.0 (1.4, 10.9)†	4.0 (1.5, 11.1)†
*40–49*	4.4 (1.7, 11.6)†	3.3 (1.1, 9.8)‡	3.4 (1.1, 10.3)‡	8.6 (3.6, 20.4)†	7.8 (2.9, 20.8)†	7.6 (2.8, 20.2)†
*50–59*	7.5 (3.0, 19.1)†	6.0 (2.0, 17.7)†	6.0 (2.0, 17.7)†	18.0 (7.8, 42.0)†	14.4 (5.4, 38.7)†	14.3 (5.4, 38.1)†
*60–69*	7.6 (3.1, 18.4)†	6.5 (2.2, 19.3)†	6.3 (2.1, 18.9)†	22.7 (10.0, 51.4)†	17.3 (6.5, 45.8)†	16.6 (6.3, 44.0)†
Sex						
*Male*	ref	ref	ref	ref	ref	ref
*Female*	1.0 (0.7, 1.5)	0.8 (0.4, 1.5)	0.8 (0.5, 1.4)	1.4 (1.1, 1.8)‡	1.2 (0.7, 1.8)	1.2 (0.8, 1.9)
Highest educational level						
*No formal schooling*	ref	ref	ref	ref	ref	ref
*Primary school completed*	0.7 (0.4, 1.2)	0.9 (0.5, 1.6)	0.9 (0.5, 1.6)	0.4 (0.3, 0.6)‡	0.8 (0.5, 1.1)	0.8 (0.6, 1.2)
*Secondary school completed*	0.6 (0.3, 0.9)‡	0.7 (0.4, 1.2)	0.7 (0.4, 1.2)	0.7 (0.5, 1.0)	1.5 (1.0, 2.3)‡	1.6 (1.1, 2.3)‡
*High school or higher education completed*	0.5 (0.2, 1.0)	0.5 (0.2, 1.1)	0.5 (0.2, 1.2)	0.4 (0.3, 0.6)‡	1.0 (0.6, 1.6)	0.9 (0.6, 1.6)
Marital status						
*Currently married*	ref	ref	ref	ref	ref	ref
*Others (never married/separated divorced/widowed)*	0.9 (0.6, 1.3)	1.6 (0.9, 2.6)	1.6 (0.9, 2.7)	0.7 (0.5, 0.9)†	1.1 (0.8, 1.5)	1.1 (0.8, 1.5)
Employment status						
*Government employee*	ref	ref	ref	ref	ref	ref
*Non-government employee*	0.4 (0.2, 0.8)‡	0.3 (0.1, 0.8)‡	0.3 (0.1, 0.7)†	0.9 (0.4, 2.0)	1.6 (0.8, 3.4)	1.6 (0.7, 3.3)
*Self-employed*	0.4 (0.2, 0.8)‡	0.3 (0.1, 0.5)§	0.2 (0.1, 0.5)§	1.2 (0.6, 2.4)	1.5 (0.7, 2.9)	1.4 (0.7, 2.8)
*Unpaid (non-paid, students, homemakers, retirees, unemployed)*	0.3 (0.1, 0.6)§	0.2 (0.1, 0.4)§	0.2 (0.1, 0.4)§	1.8 (0.9, 3.4)	1.8 (0.8, 3.8)	1.7 (0.8, 3.7)
BMI						
*Underweight (<18.5 kg/m^2^)*	0.7 (0.3, 1.6)	0.9 (0.4, 2.3)	0.9 (0.4, 2.3)	0.5 (0.2, 1.1)	0.7 (0.3, 1.7)	0.8 (0.3, 1.8)
*Normal (18.5–<23 kg/m^2^)*	ref	ref	ref	ref	ref	ref
*Overweight (23–<27.5 kg/m^2^)*	1.5 (1.0, 2.4)	1.4 (0.8, 2.5)	1.4 (0.9, 2.4)	1.7 (1.3, 2.3)§	1.4 (1.0, 1.9)	1.4 (1.0, 2.0)†
*Obese (≥27.5 kg/m^2^)*	3.2 (2.1, 4.9)§	3.1 (1.8, 5.1)§	3.1 (1.8, 5.2)§	2.5 (1.7, 3.7)§	1.7 (1.1, 2.5)‡	1.7 (1.1, 2.5)‡
Smoking tobacco product use						
*Never*	ref	ref	ref	ref	ref	ref
*Former user*	1.1 (0.6, 1.9)	0.8 (0.5, 1.5)	0.8 (0.5, 1.5)	1.2 (0.8, 1.8)	0.9 (0.6, 1.6)	0.9 (0.6, 1.6)
*Current user*	1.1 (0.6, 1.8)	1.0 (0.4, 2.2)	1.0 (0.4, 2.1)	1.0 (0.7, 1.4)	0.9 (0.6, 1.6)	1.0 (0.6, 1.6)
Smokeless tobacco product use						
*Never*	ref	ref	ref	ref	ref	ref
*Former user*	2.0 (1.0, 4.2)	1.3 (0.6, 2.9)	1.3 (0.6, 2.8)	1.6 (0.8, 2.8)	0.9 (0.6, 1.6)	0.8 (0.5, 1.4)
*Current user*	1.2 (0.7, 2.3)	0.8 (0.4, 1.5)	0.8 (0.4, 1.6)	2.4 (1.6, 3.7)§	1.0 (0.6, 1.5)	0.9 (0.6, 1.5)
Alcohol consumption						
*No*	ref	ref	ref	ref	ref	ref
*Occasionally (<once per day)*	0.7 (0.4, 1.1)	0.9 (0.5, 1.4)	0.9 (0.5, 1.4)	0.7 (0.5, 1.0)‡	1.1 (0.8, 1.5)	1.1 (0.8, 1.5)
*Daily (≥once per day)*	1.2 (0.6, 2.2)	1.3 (0.6, 3.0)	1.3 (0.5, 3.1)	1.7 (1.0, 2.9)‡	1.8 (1.1, 3.1)‡	2.0 (1.2, 3.3)‡
Sugar-sweetened beverage consumption						
*No*	ref	ref	ref	ref	ref	ref
*Occasionally (<once per day)*	0.8 (0.5, 1.3)	1.1 (0.7, 1.6)	1.1 (0.7, 1.6)	0.4 (0.3, 0.6)§	0.7 (0.5, 1.0)‡	0.8 (0.6, 1.0)
*Daily (≥once per day)*	0.5 (0.3, 0.8)†	0.7 (0.4, 1.1)	0.7 (0.4, 1.1)	0.5 (0.3, 0.7)§	0.9 (0.6, 1.3)	0.9 (0.7, 1.3)
Fruits consumption						
*Low (<1.5 servings)*	ref	ref	ref	ref	ref	ref
*Moderate (1.5–2.5 servings on average per day)*	1.1 (0.6, 2.0)	1.1 (0.7, 2.0)	1.1 (0.7, 1.9)	1.2 (0.8, 1.8)	1.1 (0.8, 1.7)	1.2 (0.8, 1.8)
*Adequate (≥2.5 servings on average per day)*	1.4 (0.9, 2.2)	1.5 (1.0, 2.2)	1.5 (1.0, 2.3)	1.0 (0.7, 1.5)	0.9 (0.6, 1.2)	0.9 (0.6, 1.3)
Vegetable consumption						
*Low (<1.5 servings)*	ref	ref	ref	ref	ref	ref
*Moderate (1.5–2.5 servings on average per day)*	1.1 (0.6, 1.9)	1.1 (0.6, 2.1)	1.1 (0.6, 2.1)	0.9 (0.6, 1.2)	1.0 (0.7, 1.4)	1.0 (0.8, 1.4)
*Adequate (≥2.5 servings on average per day)*	1.3 (0.8, 2.1)	1.2 (0.7, 2.1)	1.2 (0.7, 2.1)	1.2 (0.9, 1.7)	1.4 (1.0, 2.0)‡	1.4 (1.0, 1.9)
Physical activity						
*Low*	ref	ref	ref	ref	ref	ref
*Adequate*	2.4 (1.1, 5.3)‡	2.4 (1.1, 5.0)‡	2.4 (1.1, 5.1)‡	0.8 (0.5, 1.3)	0.8 (0.5, 1.2)	0.8 (0.5, 1.3)
Comorbid hypertension						
*No*	ref	ref	ref	ref	ref	ref
*Yes*	2.5 (1.8, 3.5)§	1.2 (0.9, 1.7)	1.2 (0.9, 1.6)	5.1 (4.0, 6.6)§	2.3 (1.7, 3.2)§	2.4 (1.8, 3.1)§
Household economic status in quantiles						
*Poorest*	1.0 (0.6, 1.7)		1.0 (0.5, 1.7)	2.1 (1.5, 3.0)§		1.6 (1.1, 2.2)†
*Poorer*	1.6 (0.9, 3.0)		1.5 (0.8, 2.8)	1.1 (0.7, 1.6)		0.9 (0.6, 1.3)
*Middle*	ref		ref	ref		ref
*Richer*	1.2 (0.7, 2.0)		1.0 (0.6, 1.7)	1.2 (0.8, 1.7)		1.0 (0.7, 1.4)
*Richest*	1.2 (0.6, 2.4)		1.0 (0.5, 2.0)	1.5 (1.0, 2.2)		1.1 (0.7, 1.5)
Place of residence						
*Rural*	ref		ref	ref		ref
*Urban*	1.2 (0.8, 1.9)		1.2 (0.8, 1.8)	1.5 (1.1, 2.0)‡		1.3 (1.0, 1.8)
Region						
*Central Plain*	ref		ref	ref		ref
*Tonle Sap*	1.1 (0.7, 1.7)		1.2 (0.8, 1.8)	0.9 (0.6, 1.3)		1.3 (1.0, 1.8)
*Coastal and sea*	0.9 (0.5, 1.7)		1.0 (0.5, 1.8)	0.5 (0.3, 1.0)†		0.7 (0.4, 1.3)
*Plateau and mountains*	0.8 (0.4, 1.6)		1.2 (0.7, 2.0)	0.7 (0.4, 1.1)		1.0 (0.7, 1.6)

BMI – body mass index, CI – confidence interval, ref – reference

*Model 1. Unadjusted model. Model 2. Adjusted for individual-level factors (participant’s age, sex, highest educational, marital status, employment status, body mass index, smoking tobacco product use, smokeless tobacco product use, alcohol consumption, sugar-sweetened beverage consumption, fruits consumption, vegetable consumption, physical activity, and comorbid hypertension. Model 3. Further adjusted for household- and contextual-factors (household income quintile, place of residence, and region).

†*P* < 0.01.

‡*P* < 0.05.

§*P* < 0.001.

**Table 3:** Risk factors for undiagnosed diabetes

Variables	Prevalence ratio (95% CI)		
	Model 1*	Model 2*	Model 3*
Age in years			
*18–29*	ref	ref	ref
*30–39*	3.1 (1.1, 8.4)†	3.6 (1.2, 10.6)†	4.1 (1.4, 11.8)‡
*40–49*	6.1 (2.4, 15.2)§	5.6 (1.9, 16.2)†	6.0 (2.1, 17.0)§
*50–59*	9.0 (3.7, 22.3)§	7.4 (2.5, 22.2)§	8.5 (2.9, 24.6)§
*60–69*	9.4 (4.0, 21.9)§	7.0 (2.5, 19.7)§	7.6 (2.8, 20.6)§
Sex			
*Male*	ref	ref	ref
*Female*	1.0 (0.7, 1.6)	0.9 (0.5, 1.6)	0.9 (0.4, 1.6)
Highest educational level			
*No formal schooling*	ref	ref	ref
*Primary school completed*	0.4 (0.2, 0.6)§	0.5 (0.3, 1.0)	0.6 (0.3, 1.1)
*Secondary school completed*	0.7 (0.4, 1.4)†	1.4 (0.7, 2.6)	1.6 (0.8, 2.9)
*High school or higher education completed*	0.4 (0.2, 0.9)	0.7 (0.3, 1.5)	0.9 (0.4, 1.7)
Marital status			
*Currently married*	ref	ref	ref
*Others (never married/separated divorced/widowed)*	0.7 (0.4, 1.1)	1.4 (0.9, 2.0)	1.4 (0.9, 2.1)
Employment status			
*Government employee*	ref	ref	ref
*Non-government employee*	1.9 (0.6, 6.4)	2.8 (1.0, 8.0)	3.0 (1.1, 8.3)†
*Self-employed*	2.0(0.7, 6.1)	2.1 (0.8, 5.5)	2.1 (0.8, 5.2)
*Unpaid (non-paid, students, homemakers, retirees, unemployed)*	2.6 (0.8, 8.1)	3.0 (1.0, 8.7)†	3.1 (1.0, 9.0)†
BMI			
*Underweight (<18.5 kg/m^2^)*	0.5 (0.2, 1.5)	0.8 (0.3, 2.3)	0.8 (0.3, 2.4)
*Normal (18.5–<23 kg/m^2^)*	ref	ref	ref
*Overweight (23–<27.5 kg/m^2^)*	1.8 (1.2, 2.8)‡	1.5 (0.9, 2.5)	1.7 (1.0, 2.7)†
*Obese (≥27.5 kg/m^2^)*	3.2 (1.9, 5.5)§	2.4 (1.3, 4.3)‡	2.6 (1.5, 4.5)§
Smoking tobacco product use			
*Never*	ref	ref	ref
*Former user*	1.4 (0.7, 2.5)	1.0 (0.4, 2.2)	1.0 (0.5, 2.2)
*Current user*	1.2 (0.7, 1.9)	0.9 (0.4, 1.9)	0.9 (0.4, 1.8)
Smokeless tobacco product use			
*Never*	ref	ref	ref
*Former user*	1.1 (0.5, 2.8)	0.8 (0.4, 1.9)	0.7 (0.3, 1.5)
*Current user*	1.9 (1.0, 3.6)†	0.9 (0.5, 1.9)	0.8 (0.4, 1.7)
Alcohol consumption			
*No*	ref	ref	ref
*Occasionally (<once per day)*	1.0 (0.6, 1.5)	1.1 (0.7, 1.7)	1.1 (0.7, 1.7)
*Daily (≥once per day)*	2.6 (1.3, 5.2)‡	2.4 (1.0, 5.6)†	2.5 (1.1, 5.6)†
Sugar-sweetened beverage consumption			
*No*	ref	ref	ref
*Occasionally (<once per day)*	0.7 (0.5, 1.2)	1.1 (0.7, 1.9)	1.2 (0.7, 2.0)
*Daily (≥once per day)*	0.9 (0.6, 1.5)	1.7 (1.0, 2.8)	1.8 (1.1, 3.1)†
Fruits consumption			
*Low (<1.5 servings)*	ref	ref	ref
*Moderate (1.5–2.5 servings on average per day)*	1.0 (0.6, 1.8)	1.0 (0.6, 1.9)	1.1 (0.6, 2.1)
*Adequate (≥2.5 servings on average per day)*	1.1 (0.6, 1.8)	0.8 (0.5, 1.4)	0.9 (0.5, 1.5)
Vegetable consumption			
*Low (<1.5 servings)*	ref	ref	ref
*Moderate (1.5–2.5 servings on average per day)*	0.7 (0.4, 1.2)	0.8 (0.4, 1.4)	0.8 (0.4, 1.4)
*Adequate (≥2.5 servings on average per day)*	1.2 (0.8, 2.0)	1.3 (0.8, 2.2)	1.2 (0.8, 1.9)
Physical activity			
*Low*	ref	ref	ref
*Adequate*	1.0 (0.5, 2.1)	1.0 (0.5, 1.8)	1.1 (0.6, 1.8)
Comorbid hypertension			
*No*	ref	ref	ref
*Yes*	4.9 (3.3, 7.4)§	2.8 (1.7, 4.5)§	2.8 (1.8, 4.5)§
Household economic status in quantiles			
*Poorest*	3.6 (2.0, 6.6)§		3.0 (1.6, 5.7)§
*Poorer*	1.8 (0.9, 3.3)		1.5 (0.8, 2.9)
*Middle*	ref		ref
*Richer*	1.4 (0.8, 2.6)		1.2 (0.6, 2.2)
*Richest*	1.9 (1.0, 3.8)		1.3 (0.6, 2.6)
Place of residence			
*Rural*	ref		ref
*Urban*	1.2 (0.7, 1.9)		1.2 (0.7, 1.8)
Region			
*Central Plain*	ref		ref
*Tonle Sap*	1.1 (0.7, 1.8)		1.6 (1.0, 2.4)
*Coastal and sea*	0.5 (0.2, 1.2)		0.5 (0.2, 1.2)
*Plateau and mountains*	0.9 (0.5, 1.8)		1.4 (0.8, 2.6)

BMI – body mass index, CI – confidence interval, ref – reference

*Model 1. Unadjusted model. Model 2. Adjusted for individual-level factors (participant’s age, sex, highest educational, marital status, employment status, body mass index, smoking tobacco product use, smokeless tobacco product use, alcohol consumption, sugar-sweetened beverage consumption, fruits consumption, vegetable consumption, physical activity, and comorbid hypertension. Model 3. Further adjusted for household- and contextual-factors (household income quintile, place of residence, and region).

†*P* < 0.05.

‡*P* < 0.01.

§*P* < 0.001.

## DISCUSSION

Utilising nationally representative data from the 2023 STEPS survey, we examined educational and economic disparities and risk factors associated with diabetes and IFG in Cambodia. Our findings showed a high prevalence of diabetes, particularly among the participants with no formal education and from the poorest households. The magnitude of educational inequality in the prevalence of diabetes was significant, with the disease concentrated among the non-educated population. The economic inequality was less pronounced, but was evident in the rural areas. Other key factors independently associated with diabetes were advanced age ≥40 years, overweight and obesity, comorbid hypertension, and daily alcohol consumption. Daily SSB consumption also showed an increased risk of undiagnosed diabetes.

In our study, the prevalence of diabetes among adults aged 18 to 69 years was 6.4%, largely consistent with the 2021 International Diabetes Federation report, which showed a modelled estimate of 5.9% among adults aged 20–70 years [2,4]. The prevalence of diabetes was the highest among participants with no formal education, and a significantly large disparity was found between the non-educated and higher-educated populations in both rural and urban areas. It is important to note that the proportion of the non-educated population in this study (58.5%) was higher than the 35.1% indicated in the 2019 Census, likely due to the higher percentage of participants aged >50 years [30]. Nevertheless, this result highlights the vital role of basic education in diabetes prevention and underscores the need to strengthen educational interventions for individuals without formal schooling. Education can equip individuals with the knowledge and skills to make appropriate health decisions and maintain good health [31]. Considering the limited literacy of people without formal schooling, it will be important to adapt communication strategies to convey information on the prevention of diabetes, for example, using visual aids and audio rather than text [32]. The source of health information of this population may also tend to be the Village Health Support Groups, who are lay health workers that play a vital role in linking the community and health centres in Cambodia. A global meta-analysis shows that, when adapted to the context, community-based educational interventions may reduce the incidence of diabetes by 46% through reductions in BMI and waist circumference [33].

This study also observed the highest prevalence of diabetes among the poorest households and the economic inequality between the poorest and the richest was particularly evident in the rural areas. Consistent with previous studies, low economic status also remained as a significant risk factor for diabetes after adjusting for individual and contextual factors [14,34,35]. The potential mechanisms that have been suggested to explain the association between low economic status and diabetes include psychosocial stress, harmful lifestyle, poor diet quality, and decreased access to regular health screening to prevent the disease [14,35,36]. In Cambodia, people identified as poor by the Ministry of Planning through the Identification of Poor Households Programme can have their health services fees at public health facilities covered by the Health Equity Fund, a social health protection scheme for the Identification of Poor Households Programme members [37]. In 2024, about 4.8 million people, or 27.4% of the total population, were enrolled in this program [38]. However, regular health screening has not been a common practice in Cambodia. Efforts are under way to expand diabetes and hypertension screening at health centres country-wide and to revise the Health Equity Fund payment rates to ensure that the poor are adequately served through the second phase of the Health Equity and Quality Improvement Project, under way since 2022 [39].

In the risk factor analysis, we identified advanced age over 40 years, overweight and obesity and comorbid hypertension as important factors associated with diabetes, consistent with global reports and previous studies from Cambodia and neighbouring countries [4,12,40,41]. In addition to these factors, daily alcohol consumption showed a significant association with diabetes. The relationship between alcohol consumption and diabetes has been a subject of debate. While some studies have reported a J-shaped relationship between daily alcohol consumption and type 2 diabetes risk – suggesting a protective effect of moderate alcohol intake – this effect has not been consistently observed in stratified analyses among Asian populations [21,42]. A similar finding was observed in a study from Thailand, where regular alcohol consumption was associated with an increased risk of type 2 diabetes in men (odds ratio = 1.8; 95% CI = 1.1, 3.0).

Furthermore, this study found that daily SSB consumption is associated with an increased risk of undiagnosed diabetes, in line with previous studies [16,43]. Multiple mechanisms have been suggested to explain the adverse effect, including increased glucose intolerance and insulin resistance induced by rapid absorption of high glycaemic load and excessive calorie intake due to lower satiety than solid foods containing the same amount of calories [16,43]. In Cambodia, it is among the youngest age groups that have the highest consumption of these products, with 15.7% of men and 10.4% of women aged 18–29 years reporting that they drink a carbonated soft drink one time per day [3]. Given that diabetes prevalence increases with age in Cambodia, the higher consumption of SSBs among the younger cohort may indicate a greater future risk of increased diabetes prevalence.

We utilised data from a national population-based study with a range of socioeconomic and behavioural information and a large sample size. To our knowledge, this is the first study that examined educational and economic disparities and comprehensively investigated the risk factors associated with diabetes and IFG in Cambodia. Despite the strengths, there are several limitations. First, diabetes and IFG were defined based on self-reported status of diabetes medication use and a single measurement of FBG, which may have underestimated the outcomes [44,45]. In actual clinical practice, two abnormal test results obtained at the same time (*e.g.* FBG and haemoglobin A1c) or at two different time points are required for diagnosis in the absence of unequivocal hyperglycaemia [45]. Second, information on potential confounders such as health insurance membership and family history of diabetes was not available from the survey. Future surveys could consider adding questions related to these factors. Lastly, the causal relationship between the covariates and outcomes cannot be established due to the cross-sectional design of this study.

## CONCLUSIONS

We found that diabetes and IFG are prevalent among adults in Cambodia, alongside significant educational and economic inequalities, particularly in the rural areas. These disparities underscore the need for targeted interventions to support non-educated and economically poor populations. Strengthening public health measures to address key risk factors, particularly alcohol and SSB consumption, is essential to curbing the growing burden of diabetes in Cambodia.

## Additional material


Online Supplementary Document
