# Supplementary material for: Educational and economic disparities and risk factors associated with diabetes and impaired fasting glucose in Cambodia: analysis of a national population-based study
Source: J Glob Health. 2025 Aug 22;15:04251. doi: 10.7189/jogh.15.04251 (PMC12371606; doi:10.7189/jogh.15.04251)
Supplement: Online Supplementary Document [file jogh-15-04251-s001.pdf]

Supplement to: Haruyama R, Rahman MS, Rahman MM, Khim SA, Moadsiri A, Chham S, Chhim S, Kol H, Phy M. Educational and economic disparities and risk factors associated with diabetes and impaired fasting glucose in Cambodia: analysis of a national population-based study. J Glob Health. 2025;15:04251.

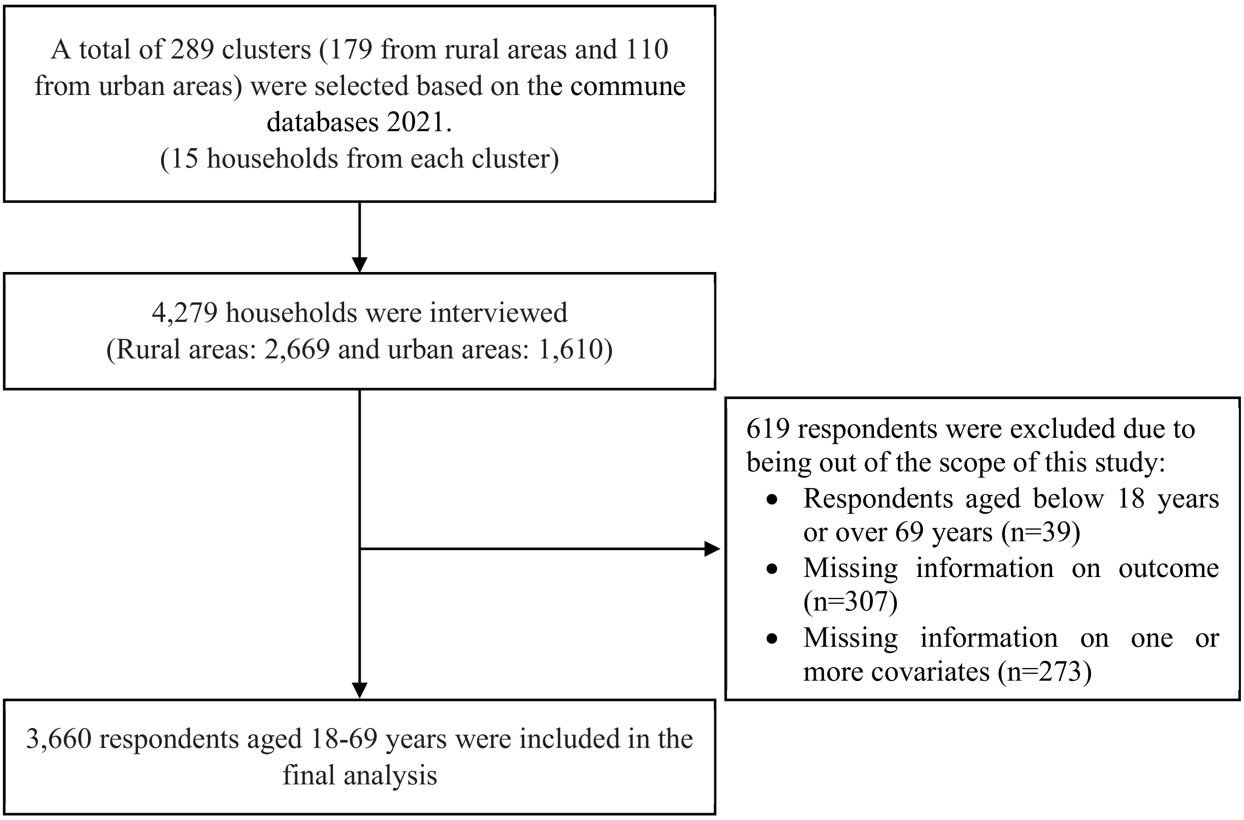

Figure S1: Participant selection flowchart

Table S1: Prevalence of diabetes and impaired fasting glucose and diabetes by individual educational status

Characteristics	Prevalence (95% confidence interval)			
	Impaired fasting glucose		Diabetes	
	No education	Higher education	No education	Higher education
National	5.5 (3.6-8.3)	2.8 (1.0-7.9)	8.9 (6.4-12.2)	3.5 (1.3-8.7)
Place of residence				
Rural	5.0 (3.0-8.2)	1.2 (0.1-10.6)	8.1 (5.5-11.8)	2.7 (0.6-11.3)
Urban	7.3 (3.4-15.0)	4.6 (1.4-14.3)	11.5 (6.3-20.1)	4.4 (1.3-14.0)

Table S2: Prevalence of diabetes and impaired fasting glucose by household economic status

Characteristics	Prevalence (95% confidence interval)			
	Impaired fasting glucose		Diabetes	
	Poorest	Richest	Poorest	Richest
National	3.5 (1.6-7.5)	4.4 (2.1-9.0)	10.0 (6.3-15.4)	6.9 (3.8-12.1)
Place of residence				
Rural	3.3 (1.4-7.8)	3.7 (1.3-10.2)	9.8 (5.9-15.8)	5.0 (2.0-11.9)
Urban	4.5 (0.8-22.2)	5.5 (1.9-14.5)	11.0 (3.6-28.9)	9.6 (4.4-19.6)

Table S3: Absolute and relative inequality in the prevalence of diabetes and impaired fasting glucose

Characteristics	Slope index of inequality (95% CI)		Relative index of inequality (95% CI)	
	Impaired fasting glucose	Diabetes	Impaired fasting glucose	Diabetes
Education-based inequality				
National	-4.4 (-8 to -0.9)*	-7.6 (-12.0 to -3.3)$	0.35 (0.10 to 1.19)	0.29 (0.10 to 0.82)*
Place of residence				
Rural	-4.7 (-9.0 to -0.3)*	-9.8 (-15.3 to -4.3)$	0.30 (0.06 to 1.49)	0.19 (0.04 to 0.82)*
Urban	-5.9 (-12.2 to 0.4)	-10.3 (-17.7 to -2.9)$	0.35 (0.05 to 2.50)	0.35 (0.08 to 1.57)
Economic inequality				
National	-0.7 (-3.7 to 2.3)	-0.7 (-4.5 to 3.0)	0.98 (0.32 to 3.00)	0.61 (0.25 to 1.52)
Place of residence				
Rural	0.1 (-3.4 to 3.5)	-4.7 (-9.1 to -0.3)*	0.91 (0.23 to 3.65)	0.31 (0.09 to 1.10)
Urban	-4.0 (-10.0 to 1.9)	-1.8 (-8.5 to 4.9)	0.90 (0.12 to 6.99)	1.03 (0.22 to 4.91)

Notes: *p<0.05, $p<0.01, §p<.001; CI, confidence interval.
